# Adolescent Acute Pancreatitis Complicated With Pseudoaneurysms and Venous Thrombosis

**DOI:** 10.7759/cureus.33228

**Published:** 2023-01-01

**Authors:** Soichiro Wada, Sasagu Matsumoto, Shuji Sai, Yoshiyasu Ambo, Yasuo Sakurai

**Affiliations:** 1 Department of Pediatrics, Teine Keijinkai Hospital, Sapporo, JPN; 2 Department of Surgery, Teine Keijinkai Hospital, Sapporo, JPN; 3 Center for Gastroenterology, Teine Keijinkai Hospital, Sapporo, JPN

**Keywords:** pancreatectomy, venous thrombosis, false aneurysms, hemorrhagic shock, acute necrotizing pancreatitis

## Abstract

Vascular complications, such as pseudoaneurysms and thrombosis, are uncommon in pediatric acute pancreatitis (AP); hence, treatment experience remains limited. Here, we report a case of adolescent AP complicated with pseudoaneurysms and venous thrombosis simultaneously. Even after multiple endovascular embolizations for pseudoaneurysms, the patient experienced hemorrhagic shock resulting from pseudoaneurysm rupture after taking anticoagulants for thrombus. Inevitably, a total pancreatectomy was performed to prevent bleeding and control local complications. In AP, even among the pediatric population, a therapeutic dilemma between bleeding prevention and anticoagulation for thrombosis may occur. Despite the lack of experience with AP and its complications, a total pancreatectomy may become an alternative therapy for refractory AP or its complications.

## Introduction

Vascular complications of acute pancreatitis (AP) include pseudoaneurysm-induced hemorrhage, ischemic complications, and hypercoagulability-induced venous thrombosis [1]. In pediatric AP, these complications are rare, and treatment experience is limited [2]. Although there are several reports of endovascular embolization for pseudoaneurysms or anticoagulation for venous thrombosis, evidence for the role of total pancreatectomy (TP) to control these complications has been rarely discussed.

In this case report, we describe an adolescent patient with AP who had concurrent complications of pancreatic pseudoaneurysms and right atrium thrombosis. Even after undergoing multiple endovascular embolizations for pseudoaneurysms, hemorrhagic shock occurred resulting from ruptured pseudoaneurysm following anticoagulation therapy initiation. TP was required to prevent bleeding and control local complications.

## Case presentation

A 13-year-old, Japanese male patient had been diagnosed with intractable ulcerative colitis (UC) for three years. Following tacrolimus therapy, he developed AP, which was suspected to be caused by a drug-induced process. Thus, tacrolimus was terminated with supportive therapy. He showed transient improvement, but AP relapsed and eventually progressed to necrotizing pancreatitis. Six months after AP onset, he was transported to our hospital for intensive care and intervention. We then provided supportive care, pain control, and intensive monitoring. He could not tolerate enteral feeding because of severe abdominal pain and hematochezia; thus, parenteral nutrition was initiated via a peripherally inserted central catheter (PICC). The tip of the PICC was located in the lower superior vena cava. Prednisolone (35 mg/day) was intravenously administered for UC remission.

Abdominal computed tomography (CT) and ultrasound showed multiple necrotic fluid collections in and around the pancreas. Gradually, these fluid collections increased in size. Three weeks after transport, these necrotic collections progressed to walled-off necrosis. We then performed endoscopic, ultrasound-guided transluminal drainage. Concurrently, he had abdominal distension caused by a large volume of pancreatic ascites, presumably caused by pancreatic duct disruption. In addition to multiple percutaneous drainages, endoscopic transpapillary pancreatic stenting (EPS) was performed to control pancreatic ascites.

**Figure 1:**
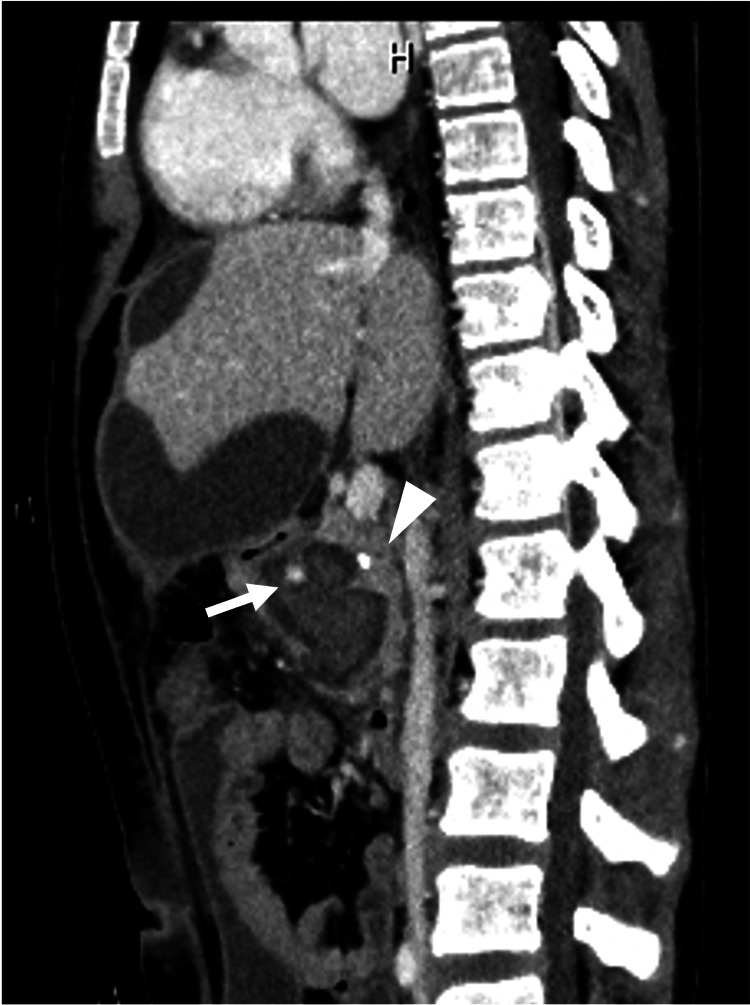
Contrast-enhanced computed tomography Pseudoaneurysm (white arrow) within the walled-off necrosis. The arrowhead shows a stent placed in the pancreatic duct.

**Figure 2:**
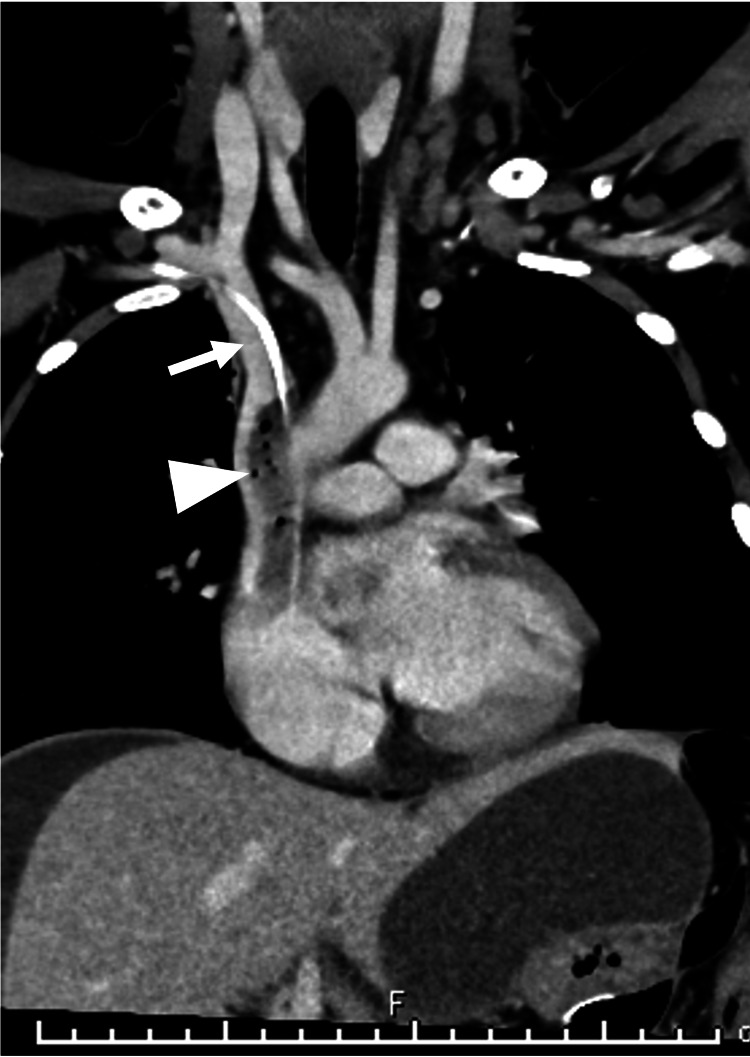
Contrast-enhanced computed tomography on the same day as the first Thrombosis (arrowhead) connected with the tip of the peripherally inserted central catheter (white arrow).

Although his abdominal pain improved after these endoscopic interventions, his high fever persisted. Two weeks later after EPS, the patient underwent contrast-enhanced CT to identify the cause of the fever. Evidence of new infection could not be detected. However, we found a pseudoaneurysm surrounded by walled-off necrosis at the pancreatic head (Figure 1) and thrombosis starting from the PICC tip to the right atrium (Figure 2).

He was immediately transferred to the angiography suite for endovascular diagnosis and intervention. Angiogram showed multiple pseudoaneurysms at three sites: gastroduodenal artery, branch of the posterior-superior pancreaticoduodenal artery, and transverse pancreatic artery. Coil embolization was subsequently performed. Regarding thrombosis, which was mobile and approximately 5 cm in size by ultrasound, we did not remove the PICC at that time because the patient still required parenteral nutrition and PICC removal may put him at risk for pulmonary embolism (PE). Two days after the first coil embolization, his abdominal pain and anemia worsened. CT scan detected a new pseudoaneurysm at the branch of the posterior-superior pancreaticoduodenal artery, as confirmed by angiography. Thus, second coil embolization was performed.

**Figure 3:**
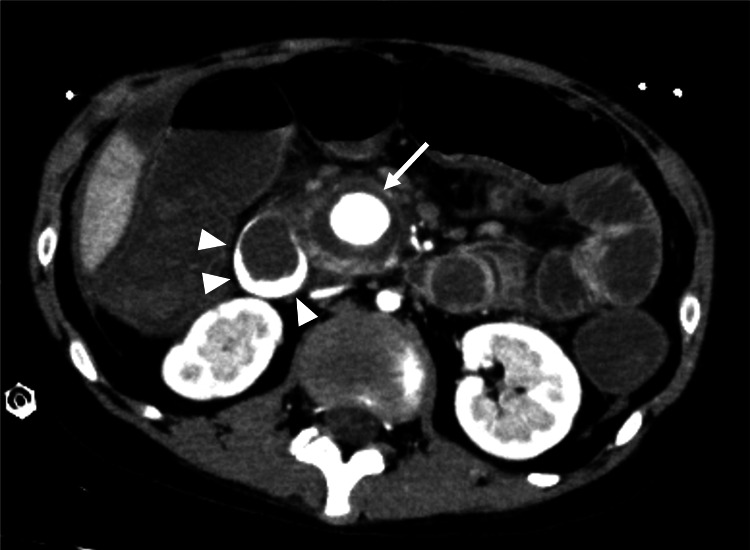
Contrast-enhanced computed tomography shows a massively enlarged pseudoaneurysm (white arrow) surrounded by a hematoma Contrast medium leaked into the duodenum (arrowheads)

After the second endovascular intervention, his symptoms improved without any recurrent pseudoaneurysm. Then, we began treating the intracardiac thrombosis with anticoagulation therapy. Three days after, 5 units/kg/hour of unfractionated heparin was modestly initiated and subsequently increased up to 7.5 units/kg/hour. Five days after heparin initiation, he suddenly complained of severe abdominal pain followed by hematemesis and massive hematochezia. His systolic blood pressure declined to 66 mmHg. An emergent CT scan revealed the development of a large pseudoaneurysm and arterial bleeding into the duodenum (Figure 3).

In addition, angiography showed a ruptured pseudoaneurysm on the branch of the anterior-inferior pancreaticoduodenal artery; this pseudoaneurysm was not detected before. We performed fluid resuscitation and a third endovascular intervention to stop the bleeding; consequently, the shock was resolved.

The risk for further bleeding should be completely reduced to treat intracardiac thrombosis safely. Therefore, we proposed total pancreatectomy (TP) to the patient and parents. Although we decided to perform TP, he complained of chest discomfort caused by PE, which was detected by CT two days preoperatively. In addition, thrombosis in the right atrium remained. After further discussion, we concluded that TP should be performed as planned because the PE was not supposed to lead to hemodynamic compromise.

Intraoperatively, continuous intravenous insulin infusion was initiated. Three days after surgery, we restarted anticoagulation therapy with heparin. Six days after surgery, enteral feeding was initiated with pancreatic enzyme replacement therapy. Subsequently, insulin was subcutaneously administered. Ten days after surgery, he developed a second PE accompanied by sepsis. Although thrombosis was still attached to the PICC tip, it was removed to control catheter-related bloodstream infection. After such an event, anticoagulation therapy was continued for two months without any PE recurrence. He was gradually able to eat and was discharged two months after TP. Intensive insulin therapy for insulin-dependent diabetes mellitus and replacement for pancreatic enzymes, microelements, and fat-soluble vitamins will be continued for the rest of his life.

## Discussion

We report a case of AP in an adolescent who then developed hemorrhagic shock after pseudoaneurysm rupture. Hemorrhage from occult pseudoaneurysms developed even after endovascular interventions and at the same time, anticoagulant therapy for PICC-related venous thrombosis was required. TP was then necessary to cease multiple refractory bleeding from pseudoaneurysms. As far as our knowledge, this is the first case report of pediatric AP complicated with pseudoaneurysms and venous thrombosis simultaneously. Furthermore, pediatric AP cases requiring TP have never been reported.

In this case, we were in a therapeutic dilemma between bleeding prevention and anticoagulation therapy. Hemorrhage is one of the vascular complications of AP and is associated with high mortality and morbidity [3,4]. Especially, pseudoaneurysms with AP require intensive management because of their vulnerability to rupture [3]. An arterial pseudoaneurysm is a ruptured vessel with a surrounding hematoma in communication with the vessel lumen. It is the result of injury to the vessel from surrounding inflammation and pancreatic enzymes [5]. Endovascular intervention for pseudoaneurysms is reportedly equally effective to surgery because of its 79%-100% efficacy [6], but rebleeding can still occur. A systematic review and meta-analysis of endovascular embolization for arterial pseudoaneurysms in pancreatitis revealed that rebleeding occurs in 98 out of 600 patients [6]. The causes of such rebleeding may be associated with the fact that pseudoaneurysms can occur at multiple sites from the time of AP diagnosis [5]. Therefore, the risk of bleeding from pseudoaneurysms remains a concern even after endovascular interventions.

Venous thrombosis is another important vascular complication of AP. Although intracardiac thrombosis with AP has not been previously reported, splanchnic vein thrombosis and deep vein thrombosis are known AP complications [1,2] that increase mortality [7,8]. Anticoagulant therapy for right atrial thrombosis or deep vein thrombosis has been recommended to reduce the risk of mortality caused by thrombus extension or embolization in a usual situation [9,10], although its safety remains controversial in AP. In a previous study, the bleeding prevalence was 12%-33% when anticoagulation therapy was applied to adult AP complicated with splanchnic vein thrombosis [11]. However, a different study concluded that anticoagulation agents appear to be safe in pediatric AP [2], although this study included only two patients treated with enoxaparin. The caveat is that venous thrombosis and PE are rare complications of AP, but they can be concomitant with pseudoaneurysms [12]. Therefore, a consensus on how to use anticoagulative agents in AP should be established, especially if the risk of developing a pseudoaneurysm is high.

In our case, we decided to perform TP to remove multiple pseudoaneurysms. TP is an operative technique involving resection of the entire pancreas along with the duodenum and spleen, followed by reconstruction. The reported 30-day mortality rate of TP for adults was as low as 1.6%-12.5% [13]. However, patients who underwent TP should be permanently treated with insulin and pancreatic enzyme replacements. Recently, islet autotransplantation has become an important option for patients with preserved insulin production when undergoing TP for benign disease [14]. However, this technique is not yet common in Japan. Other TP complications are fat-soluble vitamin deficiency and liver steatosis. Vaccination is also needed to prevent overwhelming post-splenectomy infection.

Despite these side effects, TP seemed to be the only way to control pancreatitis and its local complications in our case. Various conditions, such as chronic pancreatitis, hereditary pancreatitis, trauma, and malignant diseases, have been required for TP if they cannot be cured by limited resection. However, the clinical application for AP remains poorly discussed [13,15]. A previous study reported that among the 136 adults who underwent TP in a single institution, none had AP as the reason for TP [15]. Farkas et al. reported that TP was performed for only three out of 220 adult patients who suffered from infected pancreatic necrosis, but the details of the indications were not clearly described in this article [16]. Nevertheless, refractory AP and related complications may be controlled through TP because theoretically, they would never recur. If the local complications of AP cannot be controlled by conventional treatment and partial surgery, TP would be necessary even among the pediatric population.

## Conclusions

We had a therapeutic dilemma for an adolescent AP case to simultaneously do both hemostasis therapy for pseudoaneurysms and anticoagulation therapy for venous thrombosis. Although this situation is rare in pediatric AP, the consensus on how to use anticoagulative agents is warranted. In the limited case, TP can be an ultimate option to manage refractory AP or its complications.
